# Supplementary material for: GCN5 Is a Master Regulator of Gene Expression in the Malaria Parasite Plasmodium falciparum
Source: Cells. 2025 Jun 10;14(12):876. doi: 10.3390/cells14120876 (PMC12190302; doi:10.3390/cells14120876)
Supplement: Supplementary file 1 [file cells-14-00876-s001.zip › Supplementary Figures .pdf]

Supplementary Figures

GCN5 is a master regulator of gene expression in the malaria parasite *Plasmodium falciparum*

Amuza Byaruhanga Lucky[1], Ahmad Rushdi Shakri[1], Xiaoying Liang[1], Hui Min[1], Xiao Lian Li[1], Swamy Rakesh Adapa[2], Rays Jiang[2], Liwang Cui[1,2], Chengqi Wang[2]*, Jun Miao[1,2]*

[1] Department of Internal Medicine, Morsani College of Medicine, University of South Florida, 3720 Spectrum Blvd, Tampa, FL 33612, USA

[2] Center for Global Health and Infectious Diseases Research, College of Public Health, University of South Florida, 3720 Spectrum Blvd, Tampa, Florida 33612, USA

Short title: Gene regulation of GCN5 in the malaria parasite

A.

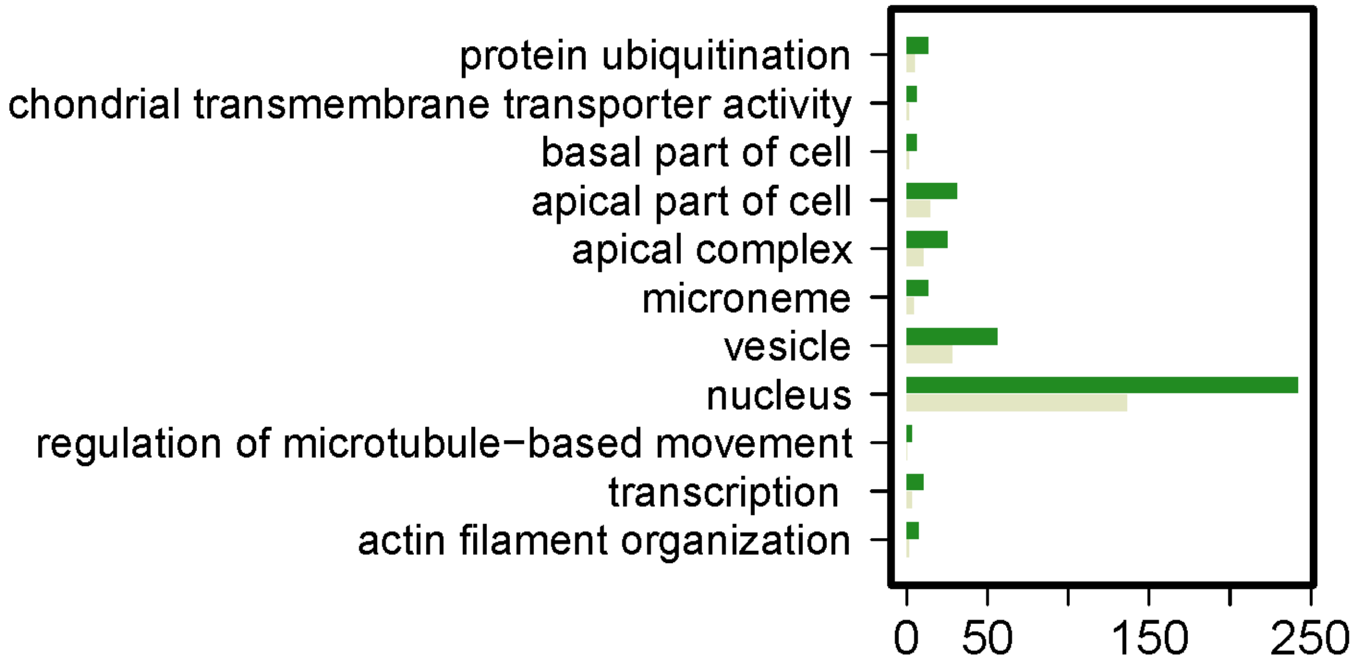

B.

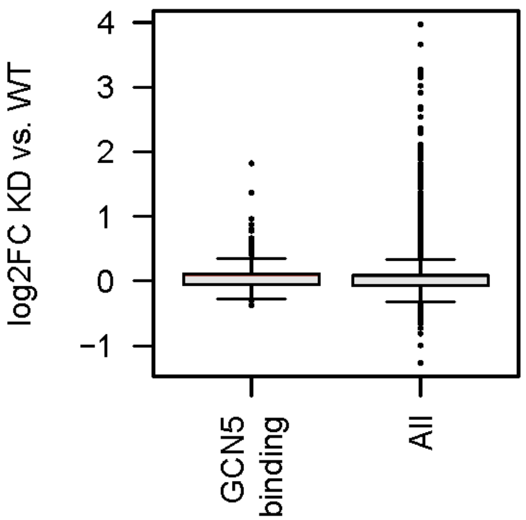

Fig. S1. Biological processes regulated by H3K9ac and the relationship between PfGCN5 coverage and expression after KD.
A. Bar graph shows GO enrichment analysis of H3K9ac enriched genes at the schizont stage, revealing many biological processes that are regulated by H3K9ac. The light and heavy green bars indicate the expected and enriched number of genes in the different pathways, respectively. **B**. PfGCN5 enriched genes (GCN5 binding) were generally not downregulated compared to other genes (All) upon PfGCN5 knockdown.

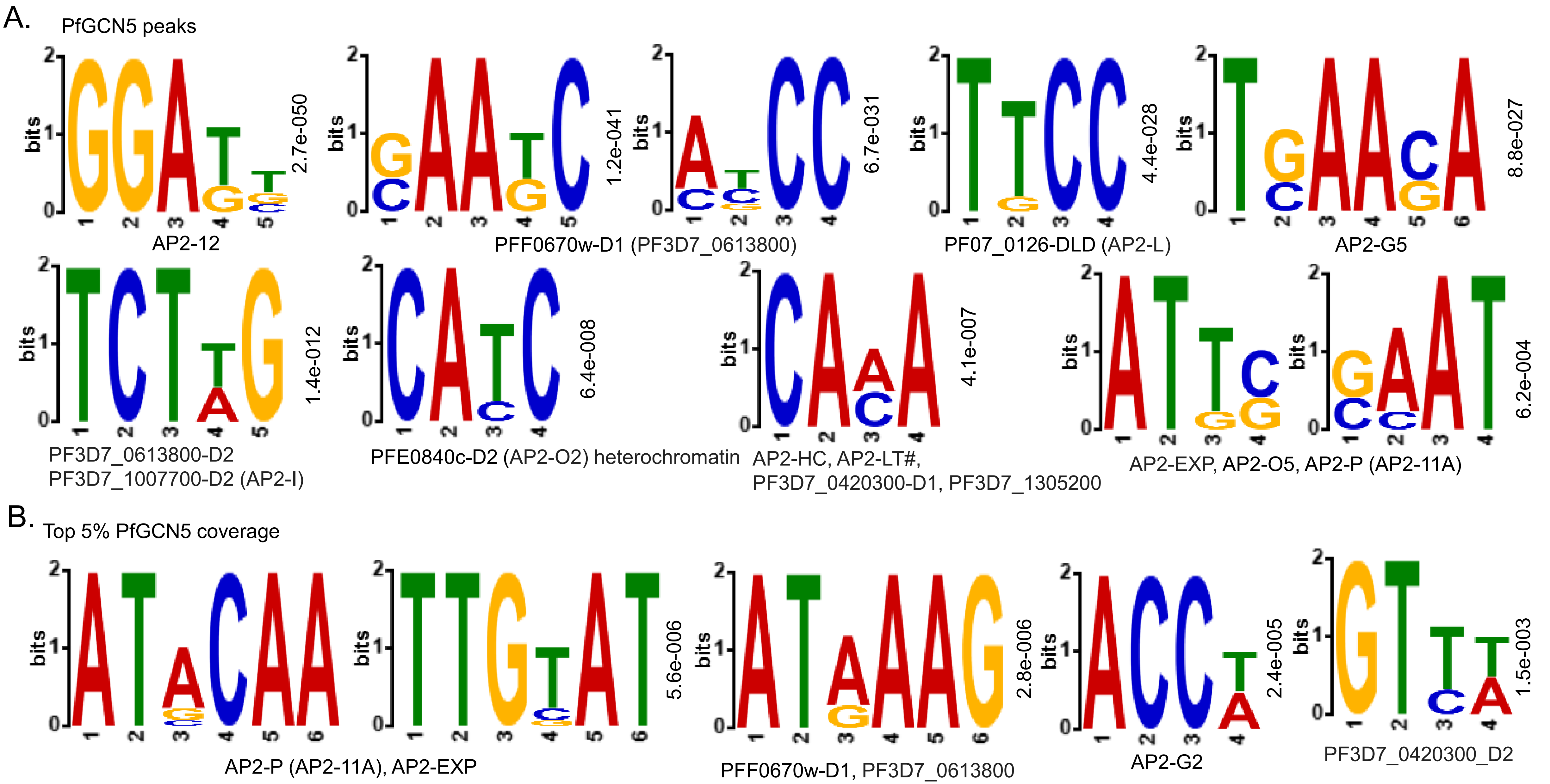

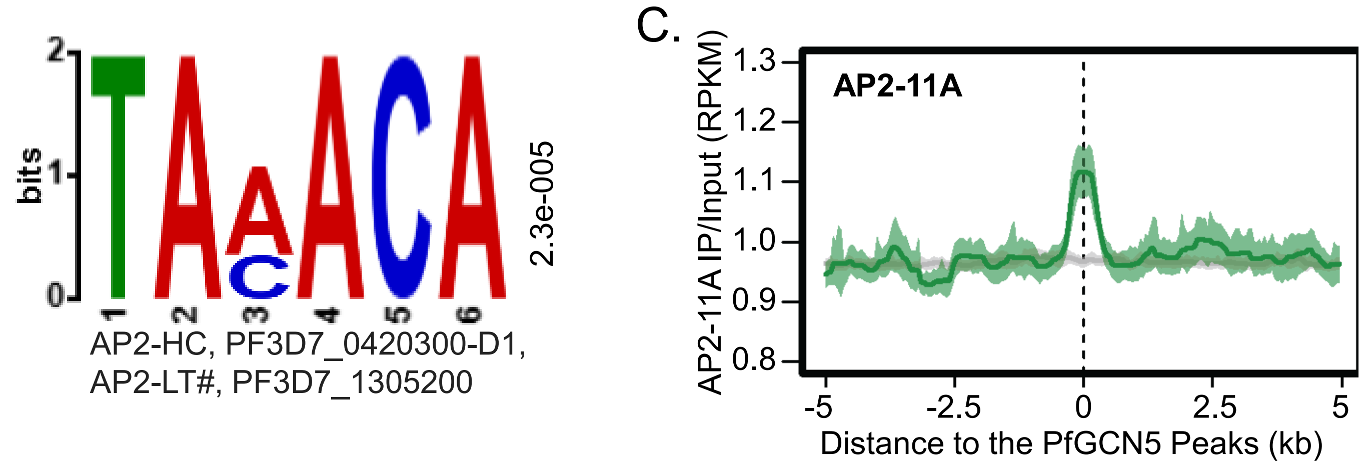

Fig. S2. Other PfGCN5 enriched motifs. **A**. Other motifs were identified in the GCN5 peaks besides the major ones in Fig. 6. **B**. Other motifs were identified in the top 5% PfGCN5 coverage in 5'UTRs besides the major ones in Fig. 6. The E-values are placed on the right side of each motif. The name of AP2(s) whose binding motifs overlap with the identified motif were listed under each motif. AP2-LT# denotes its binding motif identified by *in vitro* protein-binding microarrays (PBMs) not by *in vivo* chromatin binding motif search. **C**. PfGCN5 peaks were colocalized with the peaks of AP2-11A, which was also named AP2-P. The gray line denotes AP2-11A signals at the sites other than PfGCN5 peaks.

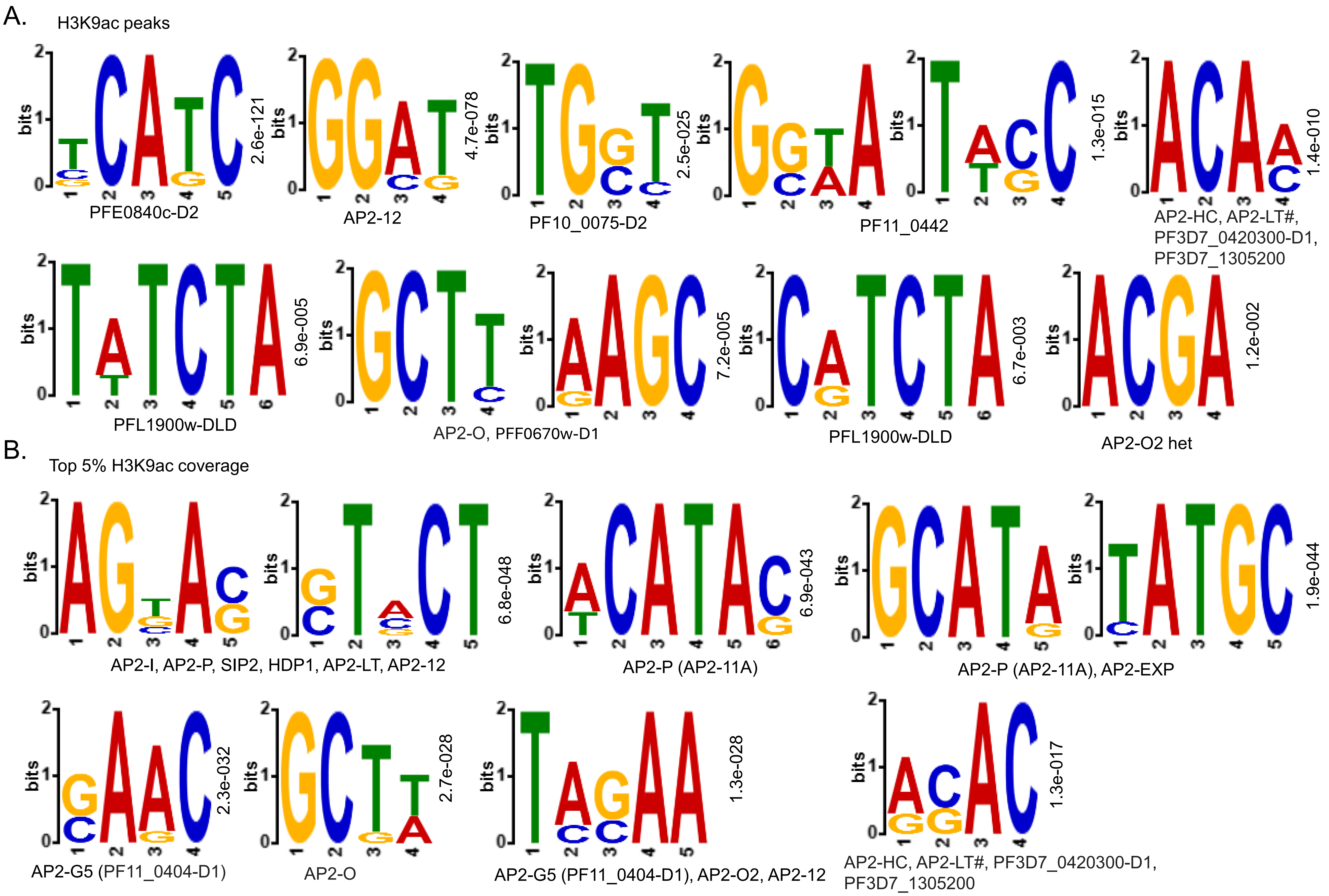

Fig. S3. H3K9ac enriched motifs. **A**. Motifs were identified in the GCN5 5'UTR peaks. **B**. Motifs were identified in the top 5% H3K9ac signals in 5'UTRs. The E-values were placed on the right sight of each motifs. The name of AP2(s) whose binding motifs overlap with the identified motif were listed under each motif. AP2-LT# denotes its binding motif identified by *in vitro* protein-binding microarrays (PBMs) not by *in vivo* chromatin binding motif search.
